# Pooled analysis of the Xpert MTB/RIF assay for diagnosing tuberculous meningitis

**DOI:** 10.1042/BSR20191312

**Published:** 2020-01-07

**Authors:** Yuan-Zhi Chen, Li-Chang Sun, Yao-Hong Wen, Zhong-Wei Li, Shu-Jin Fan, Hong-Kun Tan, Min Qiu, Zhi-Yong Pan, Qin Li, Yan-Zhen Zhao, Zhen-Xing Li, Xu-Guang Guo

**Affiliations:** 1 Department of Clinical Medicine, The Third Clinical School of Guangzhou Medical University, Guangzhou 511436, China; 2 Department of Clinical Laboratory Medicine, The Third Affiliated Hospital of Guangzhou Medical University, Guangzhou 510150, China; 3 Key Laboratory for Major Obstetric Diseases of Guangdong Province, Guangzhou 510150, China; 4 Key Laboratory of Reproduction and Genetics of Guangdong Higher Education Institutes, Guangzhou 510150, China; 5 Section of Pulmonary, Critical Care and Sleep Medicine, Department of Medicine, Yale School of Medicine, Yale University, New Haven, CT 06520–8057, U.S.A.

**Keywords:** Diagnosis, meta analysis, tuberculous meningitis, Xpert MTB/RIF

## Abstract

**Background:** Tuberculous meningitis (TBM) is one of the most serious types of extrapulmonary tuberculosis. However, low sensitivity of culture of cerebrospinal fluid (CSF) increases the difficulty in clinical diagnosis, leading to diagnostic delay, and misdiagnosis. Xpert MTB/RIF assay is a rapid and simple method to detect tuberculosis. However, the efficacy of this technique in diagnosing TBM remains unclear. Therefore, a meta-analysis was conducted to evaluate the diagnostic efficacy of Xpert MTB/RIF for TBM, which may enhance the development of early diagnosis of TBM.

**Methods:** Relevant studies in the PubMed, Embase, and Web of Science databases were retrieved using the keywords ‘Xpert MTB/RIF’, ‘tuberculous meningitis (TBM)’. The pooled sensitivity, pooled specificity, positive likelihood ratio, negative likelihood ratio, diagnostic odds ratio, summary receiver operator characteristic curve, and area under the curve (AUC) of Xpert MTB/RIF were determined and analyzed.

**Results:** A total of 162 studies were enrolled and only 14 met the criteria for meta-analysis. The overall pooled sensitivity of Xpert MTB/RIF was 63% [95% confidence interval (CI), 59–66%], while the overall pooled specificity was 98.1% (95% CI, 97.5–98.5%). The pooled values of positive likelihood ratio, negative likelihood ratio, and diagnostic odds ratio were 20.91% (12.71–52.82%), 0.40% (0.32–0.50%), and 71.49% (32.64–156.56%), respectively. The AUC was 0.76.

**Conclusions:** Xpert MTB/RIF exhibited high specificity in diagnosing TBM in CSF samples, but its sensitivity was relatively low. It is necessary to combine other high-sensitive detection methods for the early diagnosis of TBM. Moreover, the centrifugation of CSF samples was found to be beneficial in improving the sensitivity.

## Introduction

The World Health Organization (WHO) has reported tuberculosis as one of the top ten leading causes of death worldwide [1]. It is an infectious disease caused by *Mycobacterium tuberculosis (MTB)* which causes pulmonary tuberculosis and extrapulmonary tuberculosis (EPTB). More than 50% of infected people either die or become disabled [2]. Tuberculous meningitis (TBM) is a kind of central nervous system tuberculosis caused by *M. tuberculosis* invading the brain through blood circulation or other means. It belongs to a paucibacillary disease group. In the conventional culture methods, the slow growth of *M. tuberculosis* and the low sensitivity of microscopic analysis of cerebrospinal fluid (CSF) hinder the laboratory diagnosis of TBM and increase the difficulty in clinical diagnosis, leading to diagnostic delay, misdiagnosis, and increased mortality [3,4]. Therefore, the early diagnosis of TBM is of important clinical significance in reducing its harmfulness [5].

*MTB* culture is the traditional reference standard for diagnosing TBM, but it is not perfect. On the one hand, its sensitivity is moderate [6]; on the other hand, it requires strict technical operation, pollution-free environment, and long culture time. In 2010, Marais et al. [7] proposed a composite reference standard (CRS), which combined clinical assessment and laboratory examination results. However, it easily led to false-positive results due to various factors, such as the subjectivity of clinical assessment. As a fast and simple detection method, the Xpert MTB/RIF assay not only has simple operation but also has low requirement for experimental environment and can obtain results within 2 h. As Xpert MTB/RIF is tested with a sealed disposable reaction tube, the risk of contamination and the false-positive results can be reduced to almost negligible levels. However, a great controversy still exists about the diagnostic efficacy of Xpert MTB/RIF in TBM. Although several studies were conducted on the diagnostic performance of Xpert MTB/RIF test for TBM [5,8–20], the results were contradictory. Hence, this meta-analysis was performed to evaluate the diagnostic efficacy of Xpert MTB/RIF for TBM.

## Methods

### Literature retrieval

The studies published before 30 January 2019, in PubMed, Embase, and Web of Science (WOS) were searched using the following keywords: ‘Xpert MTB/RIF’, ‘tuberculous meningitis (TBM)’. The detailed search strategy is listed in ‘annex 1’. Some relevant references were also retrieved. Language restriction was set to ‘English only’.

The inclusion criteria were as follows: (1) culture or CRS (only meeting criteria on definite TBM: microbiological identification or evidence from commercial nucleic acid amplification tests of CNS MTB infection). Data were compared with Xpert MTB/RIF to determine the accuracy of diagnosing TBM; (2) human samples were analyzed; (3) enough data were generated to construct a 2 × 2 table for calculating sensitivity, specificity, and likelihood ratio, and in studies with unreported data, the authors were requested for the required data; and (4) the sample size of studies was not less than 15.

The exclusion criteria were as follows: (1) duplicate studies; (2) animal studies; (3) abstracts, conference abstracts, comments, reviews, letters, and case reports; and (4) lack of complete raw data, raw data not enough to construct 2 × 2 tables, or unable to contact authors to obtain raw data.

### Literature screening

All the studies were screened and retrieved by the two researchers independently. After completing the independent screening, two researchers checked the screening results and discussed the inclusion or exclusion of inconsistent results through consultation. If the negotiation failed, a third researcher was assigned to conduct a screening evaluation; all the results were pooled together.

### Data extraction

Two researchers independently extracted data from the enrolled studies. These data included the following: (1) the basic information (such as author, year, and region); (2) reference standard; (3) CSF centrifugation operation; (4) data included in the four-fold tables of the study, such as the total number of study samples, true positive, false positive (FP), false negative, and true negative; and (5) TBM prevalence (i.e., reference standard positive rate).

### Quality assessment of the studies

Two researchers independently evaluated the included studies using Quality assessment of diagnostic accuracy studies-2 (QUADAS-2) as a criterion [21], including Risk of bias and applicability concern. Risk of bias contains 11 signal problems, and the risk in the four domains is determined by the answers to their corresponding signal questions.

Applicability concerns include three domains. In the domain of patient’s selection, we scored ‘low concern’ if patients were evaluated at local hospitals or primary care centers. We scored ‘high concern’ if patients were evaluated exclusively as inpatients at tertiary care centers. We scored ‘unclear concern’ if the clinical setting was not reported or if information was insufficient to allow a decision.

In the domain of index test, we judged ‘low concern’ if the index test was performed as recommended by the manufacturer for sputum. We scored ‘high concern’ if the test was performed in a way that deviated from these recommendations. We scored ‘unclear concern’ if we could not tell.

In the domain of reference standard, we judged ‘high concern’ if included studies did not speciate mycobacteria isolated in culture or the patient is not a definite TBM in the CRS, ‘low concern’ if speciation was performed or the patient is a definite TBM in the CRS and ‘unclear concern’ if we could not tell.

The Review Manager 5.2 software was used to display the quality of studies and make charts. Any disagreement arising from this process was communicated to a third researcher, and a consensus was reached.

### Data analysis

The Meta-Disc software v.1.4 [22] was used to analyze the extracted four-fold table data: sensitivity, specificity, positive likelihood ratio, negative likelihood ratio, diagnostic odds ratio, and its corresponding 95% confidence interval (CI). The accuracy of TBM diagnosis by Xpert MTB/RIF was analyzed by the stochastic effect model and presented in the form of a forest map. Deeks’ funnel plot was drawn using Stata software. Deeks’ funnel plot is mainly used to observe whether there are biases in the results of a systematic evaluation or meta-analysis, such as publication bias or other biases. If the test results were *P*<0.01, the publication bias test results were significant.

### Heterogeneity analysis

Heterogeneity may arise from contingent opportunistic factors or from the threshold effect of defining negative and positive results. The heterogeneity caused by opportunistic factors was explored through the following operations: (1) visual inspection of forest maps to observe the deviation between the sensitivity and specificity of each entry and the vertical lines of the corresponding pooled values; a large deviation between the study and the line indicated a possible source of heterogeneity. (2) The chi-square *P*-value generated automatically by the Meta-Disc software was used to judge the data. A lower chi-square *P*-value indicated that heterogeneity originated from many sources and was not clear. (3) Quantitative indicators of heterogeneity were judged by the inconsistency index (*I*-square) automatically generated by the Meta-Disc software. The inconsistency index was interpreted as follows: 0–40%: low heterogeneity; 50–70%: moderate heterogeneity; and >70%: significant heterogeneity [23].

The heterogeneity caused by the threshold effect was explored by drawing summary receiver operating characteristic (SROC) curves of Xpert MTB/RIF to evaluate whether the points in the curve had a curved (shoulder-arm) pattern. Typical ‘shoulder-arm’ patterns indicated the presence of a threshold effect [22]. The Meta-Disc software automatically calculated and displayed the area under SROC curve and the Cochrane index (Q*). Cochrane index, as well as SROC curve, which is a comprehensive index reflecting continuous variables of sensitivity and specificity, reveals the relationship between sensitivity and specificity by composition. As a further evaluation of the threshold effect, the Spearman correlation coefficient was also calculated. Spearman correlation coefficient is a nonparametric measure of the dependence of two variables. It uses monotone equation to evaluate the correlation between two statistical variables. If the Spearman correlation coefficient was more than 0.6, the possibility of threshold effect was indicated. If the value was less than 0.6, the absence of threshold effect was indicated.

## Results

### Literature screening and inclusion process

**Figure 1:**
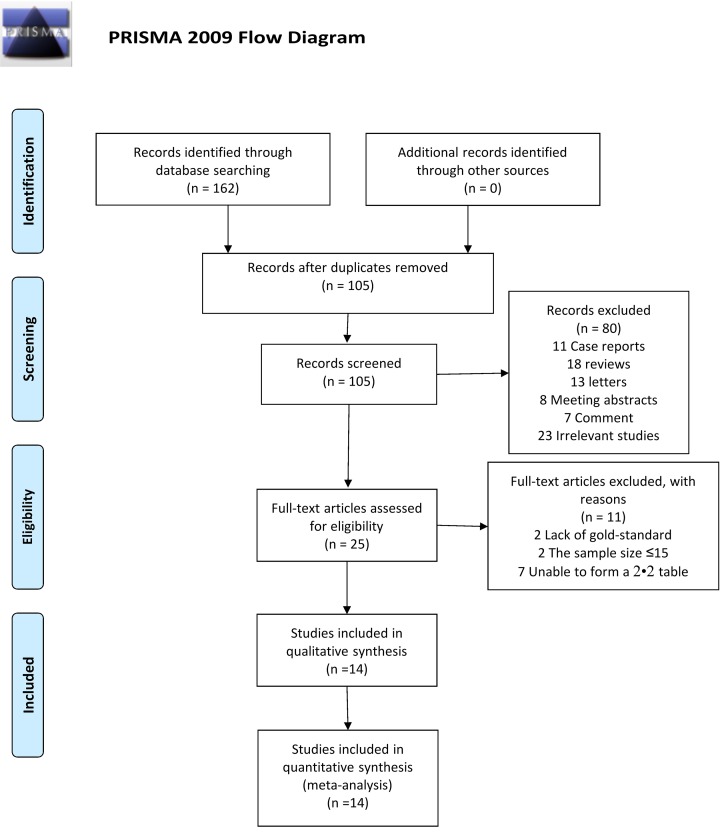
Flow diagram of study identification and inclusion *From*: Moher D., Liberati A., Tetzlaff J., Altman D.G. and The PRISMA Group (2009) *P*referred *R*eporting /tems for *S*ystematic Reviews and *M*eta-Analyses: The PRISMA Statement. *PLoS Med.***6**(7), e1000097, doi:10.1371/journal.pmed1000097

A total of 162 studies (44 in PubMed, 45 in Embase, and 73 in WOS) were searched. Of these, 57 duplicate studies were excluded. Of the remaining 105 studies, 80 studies were excluded (11 case reports, 18 reviews, 13 letters, 8 meeting abstracts, 7 comments, and 23 unrelated studies) by browsing the titles and abstracts of the studies according to the inclusion/exclusion criteria. By browsing the full text of the remaining 25 studies, 11 were further excluded because of the following reasons: 2 studies lacked the reference standard such as culture or CRS, the sample sizes of 2 studies were less than 15, and the original data of 7 studies could not form a complete 2 × 2 table. Zero gray literature was found during the second screening and the full-text browsing. Finally, 14 studies that met the inclusion/exclusion criteria were included (Figure 1).

### Characteristics and quality assessment of included studies

A total of 14 studies and 4147 samples were included, and 20 complete 2 × 2 tables were extracted from them. We extracted 20 sets of data from these 14 studies, of which 7 using CRS as a reference standard and 13 using culture as a reference standard. In addition, we divided these studies into CSF centrifugation group (15 sets of data), non-centrifugation group (4 sets of data) and mixed group (1 set of data, which contains the data of both centrifuged and non-centrifuged CSF sample). The features included in the study are summarized in ‘Table 1’.

**Table 1:** Characteristics of the included studies

Author	Year	Country	Reference standard	CSF centrifugation	Included patients, *n*	TP	FP	FN	TN	Prevalence rate (*n*%)	Prevalence rate ≥ 30%
Bahr	2015	Uganda	CRS	Yes	95	13	0	7	75	21	No
Bahr	2017	Uganda	CRS	Yes	129	10	0	13	106	18	No
Bahr	2017	Uganda	Culture	Yes	129	6	4	4	115	8	No
Cresswell	2018	Uganda	Culture	Yes	118	17	17	22	62	33	Yes
Heemskerk	2018	Vietnam; South Africa; Indonesia	Culture	Yes	602	95	0	24	483	20	No
Metcalf	2018	Peru	CRS	Yes	15	7	0	1	7	53	Yes
Metcalf	2018	Peru	Culture	Yes	37	6	1	1	29	19	No
Nhu	2013	Vietnam	CRS	Yes	379	108	1	43	196	40	Yes
Patel	2013	South Africa	Culture	Yes	46	22	1	5	18	59	Yes
Patel	2013	South Africa	Culture	NO	85	20	3	19	43	46	Yes
Patel	2013	South Africa	Culture	Yes and No^1^	119	36	4	18	61	45	Yes
Patel	2014	South Africa	CRS	No	84	15	3	16	50	37	Yes
Pink	2015	United Kingdom	Culture	Yes	740	20	13	17	690	5	No
Pink	2015	United Kingdom	Culture	Yes	735	25	3	20	687	6	No
Rufai	2017	India	Culture	No	261	27	11	22	201	19	No
Sharma	2018	Northern India	Culture	Yes	125	61	0	19	45	64	Yes
Solomons	2015	South Africa	CRS	Yes	59	5	0	8	46	22	No
Solomons	2016	South Africa	Culture	No	35	14	0	1	20	43	Yes
Wang	2016	China	CRS	Yes	153	38	0	66	49	68	Yes
Wang	2016	China	Culture	Yes	201	8	2	5	186	6	No

^1^: Indicates that the sample in the data contain samples for centrifugation and non-centrifugation; FN, false negative; TN, true negative; TP, true positive.

**Figure 2:**
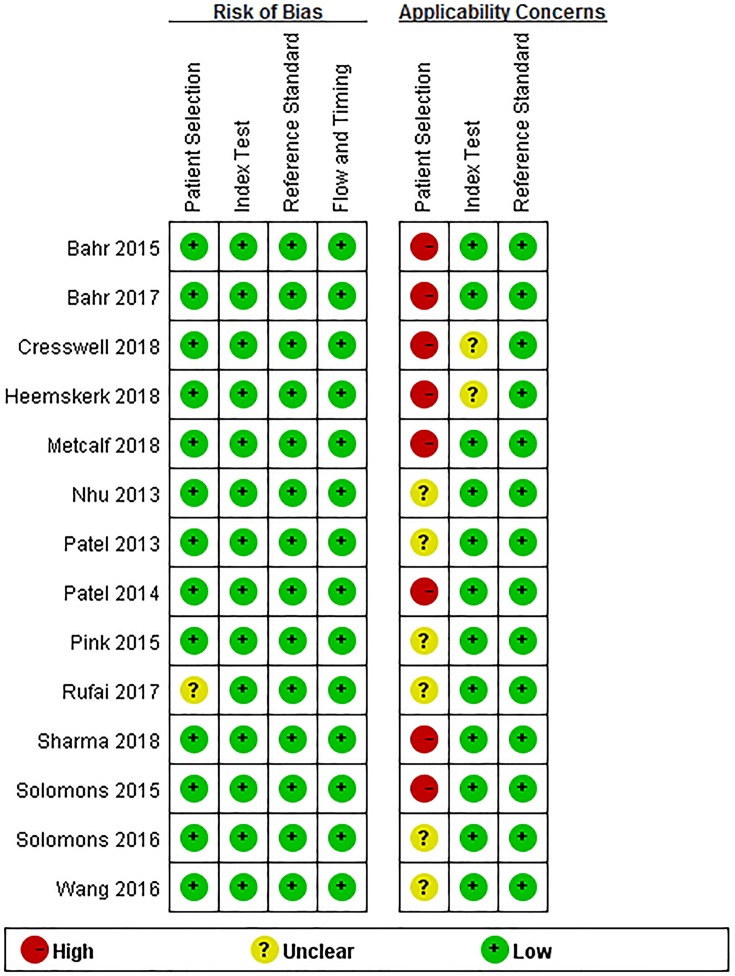
Quality evaluation of the included studies

**Figure 3:**
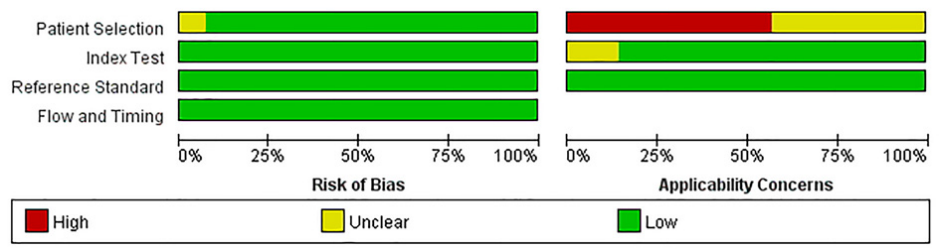
Risk of bias and applicability concerns graph: review authors’ judgments about each domain presented as percentages across the included studies

‘Figures 2 and 3’ show the quality assessment of 14 included studies, including bias risk and applicability concerns. In the patient selection domain, 13 studies (92.85%) had a lower risk of bias and 1 study (7.15%) was rated as having ‘unclear’ risk of bias because patient selection was not clear. The applicability concerns of eight studies (57.14%) were rated as ‘high concern’ because their patients were evaluated in tertiary hospital centers rather than in local community hospitals or in primary hospitals. Six studies (42.86%) were rated as ‘unknown concerns’ because it was not clear whether their patients were assessed in local primary hospitals or tertiary hospitals. In the index test domain, all the bias risks included in the studies were rated as ‘low risk’; and the applicability concerns of two studies (14.25%) were rated as ‘unknown concern’ because the details of sample processing were not reported, and the rest were ‘low concern’. In the reference standard domain, all the bias risks included in the studies were rated ‘low risk’ and their applicability concerns were ‘low concern’. In the flow and timing domain, all the studies were judged to be a low-bias risk because all patients were included in the analysis using appropriate reference criteria and with appropriate intervals between index tests and reference criteria.

### Publication bias

**Figure 4:**
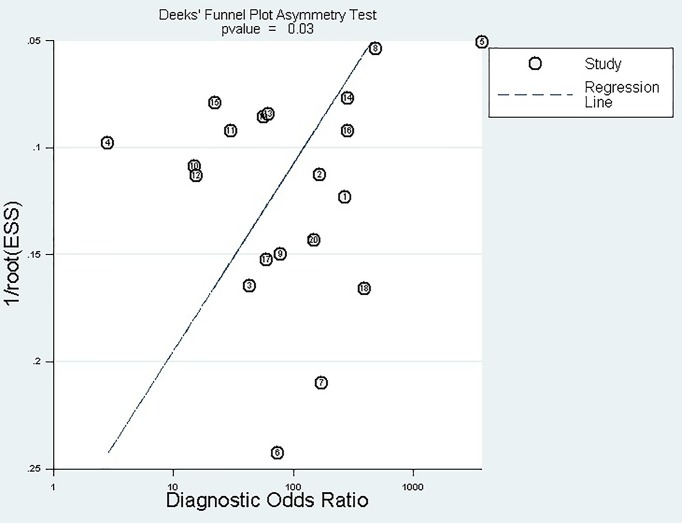
Deeks’ funnel plot asymmetry test to assess publication bias in estimates of diagnostic odds ratio for Xpert MTB/RIF detection of TBM

No publication bias was found in the Deeks’ funnel plot (Figure 4). The experimental results *P*=0.029 (>0.01) indicated no publication bias.

### Spearman correlation coefficient for threshold effect analysis

**Figure 5:**
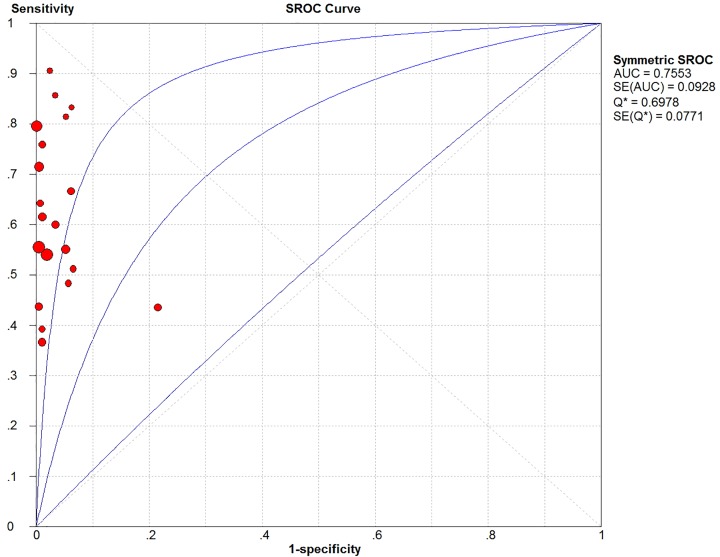
SROC curves of TBM detected by Xpert MTB/RIF

In the threshold effect analysis, the Spearman correlation coefficient was 0.014 (<0.6) and *P*-value was 0.952 (>0.05). In addition, the SROC curve was analyzed (Figure 5), which had no ‘shoulder-arm‘ distribution characteristics. Therefore, it was concluded that no threshold effect existed in the included studies.

### SROC curve

The area under the SROC curve (Figure 5) and the Cochrane (Q*) index was 0.76 and 0.70, respectively, indicating that TBM detection by Xpert MTB/RIF had relatively moderate accuracy.

### Meta-analysis results

**Figure 6:**
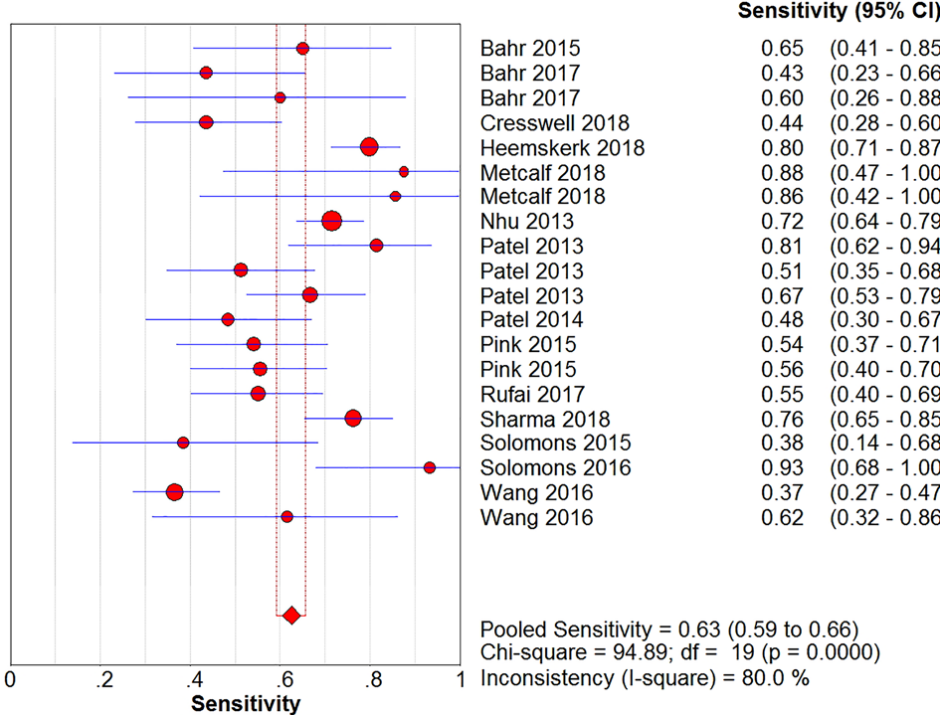
Forest plots for the pooled sensitivity of Xpert MTB/RIF

**Figure 7:**
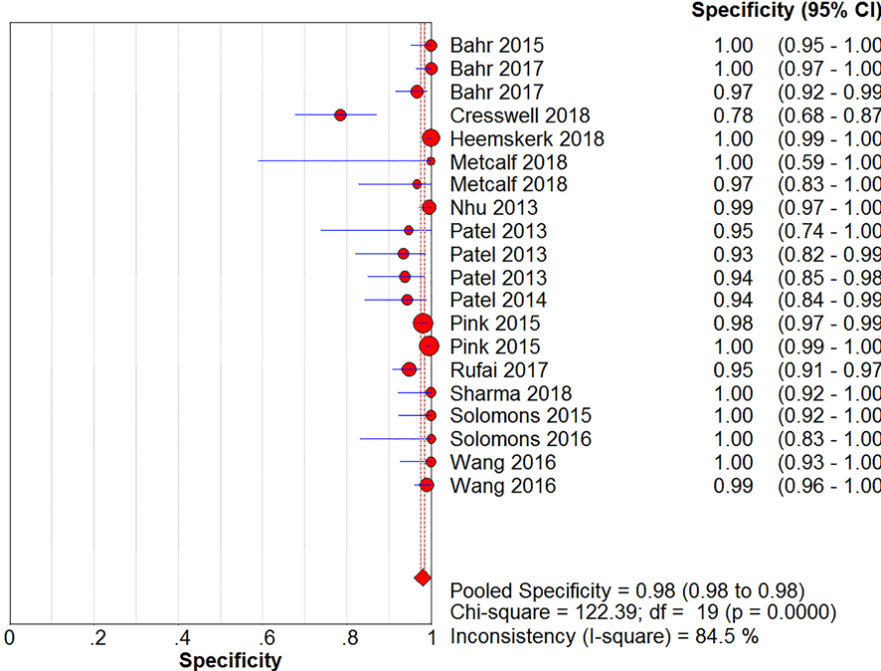
Forest plots for the pooled specificity of Xpert MTB/RIF

**Figure 8:**
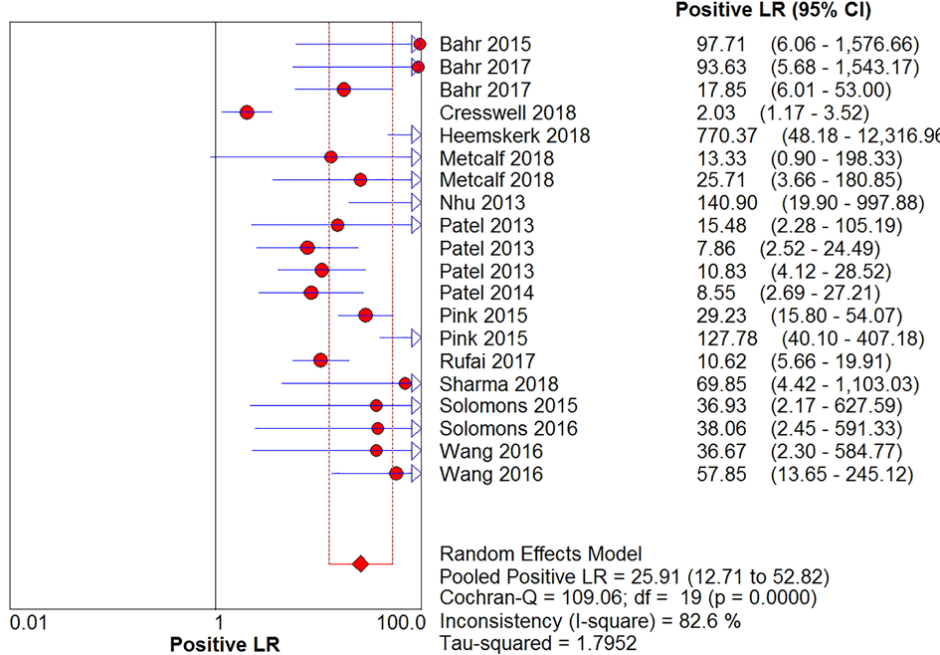
Forest plots for the pooled positive likelihood ratio of Xpert MTB/RIF

**Figure 9:**
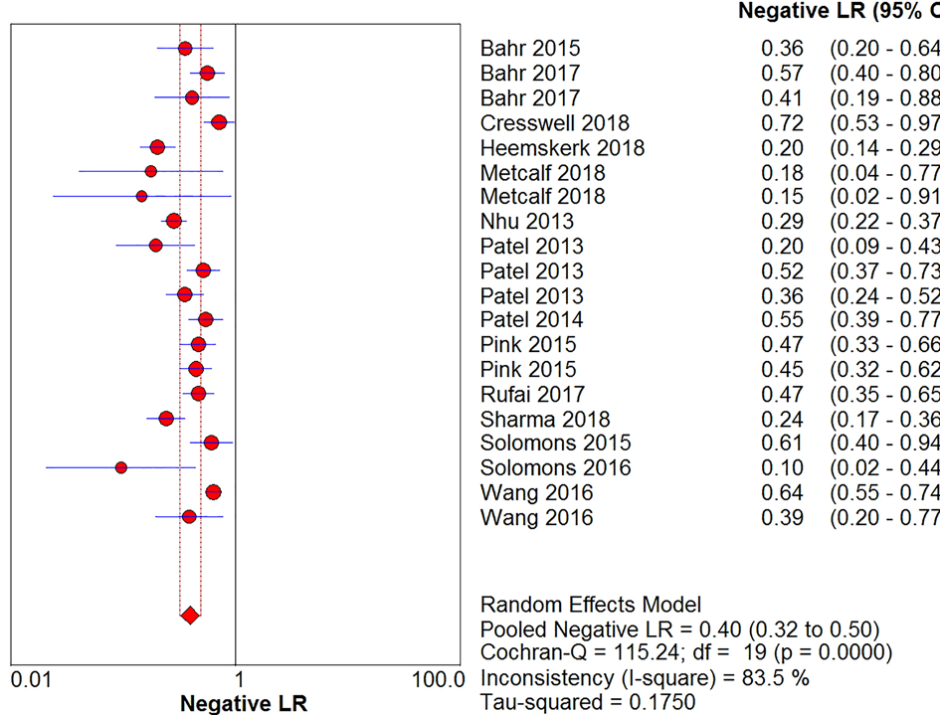
Forest plots for the pooled negative likelihood ratio of Xpert MTB/RIF

**Figure 10:**
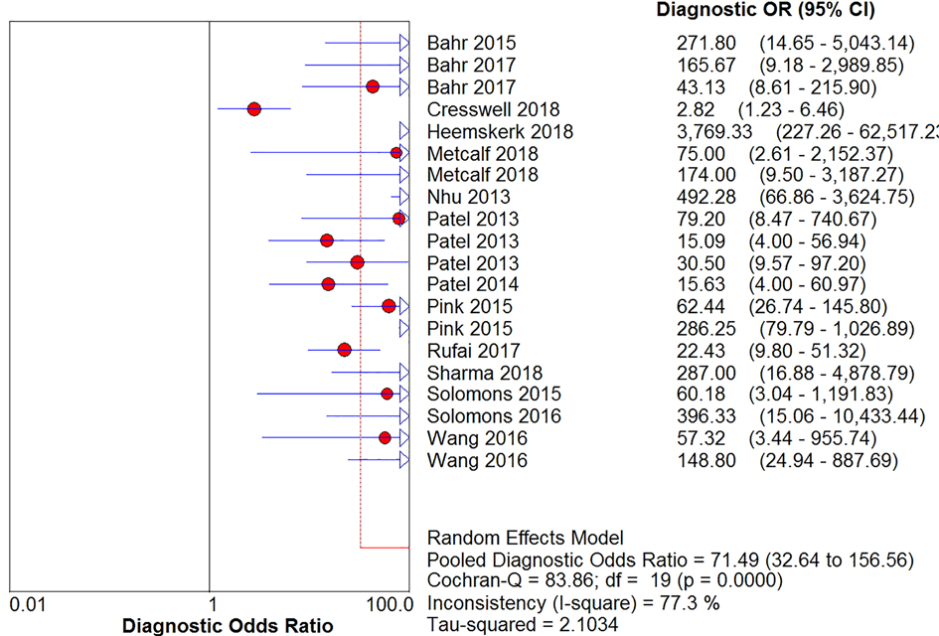
Forest plots for the pooled diagnostic odds ratio of Xpert MTB/RIF

The sensitivity, specificity, positive likelihood ratio, negative likelihood ratio, and diagnostic odds ratio (Figure 6, Figure 7, Figure 8, Figure 9 and Figure 10) and their 95% CIs were 63% (59–66%), 98.1% (97.5–98.5%), 20.91% (12.71–52.82%), 0.40% (0.32–0.50%), and 71.49% (32.64–156.56%), respectively. The results of the pooled values suggested that the accuracy of TBM detection by Xpert MTB/RIF was relatively moderate.

Overall, visual forest maps showed a large deviation between the sensitivity of more than half of the included documents and the vertical line of the pooled values, indicating a greater possibility of heterogeneity. In terms of specificity, only one document deviated from the pooled value greatly, presumably due to some unknown contingency factors, while the rest were mostly concentrated near the pooled value. The chi-square *P*-values of all the pooled values were very low, and the *I*-square values were higher than 70%.

### Subgroup analysis

Subgroup analysis was performed (Table 2) according to whether the standard reference was different (group A); whether CSF samples were centrifuged (group B); and whether the prevalence of TBM was more than 30% (i.e., positive rate; group C).

**Table 2:** Subgroup analysis results

Subgroup analysis	Sensitivity (95% CI)	Specificity (95% CI)	PLR (95% CI)	NLR (95% CI)	DOR (95% CI)	SROC	
						AUC	Q*
GROUP A							
Culture	0.67 (0.63–0.71)	0.98 (0.97–0.98)	22.45 (9.69–52.16)	0.37 (0.28–0.48)	65.73 (25.03–72.62)	0.7204	0.6693
CRS	0.56 (0.51–0.61)	0.99 (0.98–1.00)	35.05 (11.21–109.62)	0.46 (0.32–0.67)	86.47 (25.54–292.75)	0.8592	0.7899
GROUP B							
Yes	0.63 (0.60–0.67)	0.99 (0.98–0.99)	39.11 (13.70–111.64)	0.39 (0.29–0.51)	111.17 (36.80–335.87)	0.7895	0.7267
No	0.57 (0.48–0.65)	0.95 (0.92–0.97)	10.06 (6.17–16.41)	0.49 (0.37–0.64)	21.41 (10.55–43.44)	0.9985	0.9876
GROUP C							
Yes	0.62 (0.57–0.66)	0.95 (0.93–0.97)	15.24 (4.88–47.55)	0.38 (0.27–0.54)	44.42 (12.85–153.56)	0.8352	0.7674
No	0.64 (0.59–0.69)	0.99 (0.98–0.99)	42.54 (18.54–97.63)	0.42 (0.32–0.54)	0.42 (0.32–0.54)	0.6661	0.6262

Abbreviations: AUC, area under the curve; DOR, diagnostic odds ratio; NLR, negative likelihood ratio; PLR, positive likelihood ratio.

Group A: When culture was used as the reference standard, the pooled sensitivity was 67% (63–71%) and the pooled specificity was 98% (97–98%). When CRS was used as the reference standard, the pooled sensitivity was 56% (51–61%) and the pooled specificity was 99% (99–100%).

Group B: When CSF samples were centrifuged, the pooled sensitivity was 63% (60–67%) and the pooled specificity was 99% (98–99%). When CSF samples were not centrifuged, the pooled sensitivity was 57% (48–65%) and the pooled specificity was 95% (92–97%).

Group C: When the prevalence of TBM was more than 30%, the pooled sensitivity was 62% (57–66%) and the pooled specificity was 95% (93–97%). When the prevalence of TBM was less than 30%, the pooled sensitivity was 64% (59–69%) and the pooled specificity was 99% (98–99%).

## Discussion

The present study showed that the pooled specificity of Xpert MTB/RIF in diagnosing TBM was particularly high (98%), indicating that the possibility of misdiagnosis of TBM was low. The pooled sensitivity was 63% (59–66%), which was not ideal and unlikely to greatly improve the accuracy of early diagnosis of TBM.

Comparing the data of the two groups with culture and CRS as the reference standards, it was found that although the pooled specificity of the two groups was not significantly different (98 and 99%, respectively, −1%), the pooled sensitivity of the two groups was quite different (67 and 56%, 11%). The decrease in sensitivity indicated that culture might have FP results, increasing the positive results, or that culture cannot be used as a single reference standard. This coincided with the conclusions of the WHO Steering Group (Gilpin et al.) [24]. When it comes to the discussion on Publication bias, there are two arguments about the defined value of the *P*-value, one is 0.01 and the other is 0.05, depending on the accuracy of the decision. We made Deeks’ funnel plot by using Stata software. It is a default setting in the stata software: when the *P*-value is greater than 0.01, there is no publication bias. The *P*-value of our funnel plot is 0.029 (>0.01), indicating no publication bias. However, it is generally believed that when *P*<0.01, publication bias is very significant, and when *P*>0.05, it indicates that publication bias does not exist. When *P* is between 0.01 and 0.05, it can be considered that there is a certain publication bias. So we did a sensitivity analysis by manually deleting the documents one by one. With each of the studies individually removed, the corresponding pooled values and the I-square were not materially altered for all models. It indicated that no single study influenced the pooled value and the I-square qualitatively, suggesting that the results of this meta-analysis are stable.

With a culture as the reference standard, the sensitivity was 12.5% lower than that indicated by Gilpin et al. [24] (79.5%, 62.0–90.2%), although the specificity of the present study was similar (98.6%, 95.8–99.6%). The reasons might be as follows: (1) compared with the data before 2013, the present study included more studies published in the last 6 years. (2) The inclusion and exclusion criteria defined in the present study had two requirements: the sample size should be more than 15, and the four-fold table should be complete (i.e., only when at most ones of the four data, true positive, FP, true negative, false negative, are 0, that make sense). Gilpin et al. [24] included a lot of studies that did not meet the aforementioned conditions, leading to the inclusion of part of the studies and hence a smaller number of samples, besides the lack of sensitivity and specificity. It was believed that such an operation would increase the risk of experimental bias, which was not explained clearly by Gilpin et al [24].

When using CRS as the reference standard, the pooled sensitivity and specificity of the present study were not significantly different from those of Gilpin et al. [24] (0.5 and 4%, respectively). That is, CRS as the reference standard might lead to an increase in the number of positive people, thus reducing the sensitivity of Xpert MTB/RIF.

In 2018, Kohli et al. [25] reviewed the diagnostic efficacy of Xpert MTB/RIF for EPTB. They mentioned that the pooled sensitivity and 95% CI of Xpert MTB/RIF for TBM (taking culture as the reference standard) were 71.1% and 60.9 –80.4%, and the pooled specificity was 98.0% (97.0–98.8%). The pooled specificity of the two methods was the same, and the pooled sensitivity was 4.1%. The reasons for this were the same as the aforementioned differences from the study by Gilpin et al [24].

When CSF specimens were centrifuged, both sensitivity and specificity were improved by 6 and 4%, respectively. This suggested that the centrifugation of CSF samples could improve the sensitivity of Xpert MTB/RIF, which might be due to the concentration of *M. tuberculosis* by centrifugation.

The difference in the pooled susceptibility and specificity of Xpert MTB/RIF between low- and high-TBM epidemic areas was not significant, indicating that the prevalence of TBM might not be the source of heterogeneity.

Our study has the following limitations: first, clinical data were still relatively small, and the impact of unpublished positive results on the overall results could not be ruled out. In addition, few studies included the age and human immunodeficiency virus status of patients, and it was uncertain what impact it would have on the pooled values. Moreover, the impact of other potential confounding factors on the results was not explored. Finally, things in studies such as ‘patient selection’ and ‘whether the hospitals were primary or tertiary’ were unclear without contacting the authors, so the characteristics and quality were not well illustrated.

In conclusion, Xpert MTB/RIF exhibited high specificity in diagnosing TBM in CSF samples; however, its sensitivity was relatively low. It is necessary to combine other sensitive detection methods for the early diagnosis of TBM. Moreover, the centrifugation of CSF samples was found to be beneficial in improving sensitivity. However, more clinical data are needed to support the conclusions.

## Availability of Data and Materials

All data generated or analyzed during the present study are included in this published article and its supplementary information files.

## Abbreviations

CI: confidence interval
CRS: composite reference standard
CSF: cerebrospinal fluid
EPTB: extrapulmonary tuberculosis
FP: false positive
MTB: *Mycobacterium tuberculosis*
SROC: summary receiver operating characteristic
TBM: tuberculous meningitis
WHO: World Health Organization
WOS: Web of Science
